# THE IMPORTANCE OF TRANSPARENCY AND OPEN SCIENCE IN GERONTOLOGY: PERSPECTIVES FROM EARLY-CAREER RESEARCHERS

**DOI:** 10.1093/geroni/igz038.086

**Published:** 2019-11-08

**Authors:** Jennifer Lodi-Smith, Eileen K Graham

**Affiliations:** 1 Canisius College, Buffalo, New York, United States; 2 Northwestern University, Chicago, Illinois, United States

## Abstract

The past decade has seen rapid growth in conversations around and progress towards fostering a more transparent, open, and cumulative science. Best practices are being codified and established across fields relevant to gerontology from cancer science to psychological science. Many of the areas currently under development are of particular relevance to gerontologists such as best practices in balancing open science with participant confidentiality or best practices for preregistering archival, longitudinal data analysis. The present panel showcases one of the particular strengths of the open science movement - the contribution that early career researchers are making to these ongoing conversations on best practices. Early career researchers have the opportunity to blend their expertise with technology, their knowledge of their disciplines, and their vision for the future in shaping these conversations. In this panel, three early career researchers share their insights. Pfund presents an introduction to preregistration and the value of preregistration from the perspective of “growing up” within the open science movement. Seaman discusses efforts in and tool for transparency and reproducibility in neuroimaging of aging research. Ludwig introduces the idea of registered reports as a particularly useful form of publication for researchers who use longitudinal methods, and/or those who work with hard-to-access samples. The symposium will include time for the audience to engage the panel in questions and discussion about current efforts in and future directions for transparent, open, and cumulative science efforts in gerontology.

